# Implementing the new WHO guidelines on HIV post‐exposure prophylaxis: perspectives from five African countries

**DOI:** 10.1002/jia2.26447

**Published:** 2025-06-26

**Authors:** Sarah Magni, Daniel Byamukama, Maryam Sani Haske, Jane Mukami, Idah Moyo, Judith D. Auerbach

**Affiliations:** ^1^ Genesis Analytics Johannesburg South Africa; ^2^ HIV Prevention Uganda AIDS Commission Kampala Uganda; ^3^ Community Prevention and Care Services National Agency for the Control of AIDS Abuja Nigeria; ^4^ Preventive and Curative Department National Syndemic Disease Control Council Nairobi Kenya; ^5^ Ministry of Health and Child Care Harare Zimbabwe; ^6^ Department of Medicine University of California San Francisco San Francisco California USA

**Keywords:** HIV PEP, post‐exposure prophylaxis, HIV prevention, World Health Organization, global guidance, PEP to PrEP

## Abstract

**Introduction:**

Post‐exposure prophylaxis (PEP) is an important component of comprehensive HIV prevention, yet its uptake has been suboptimal globally. In July 2024, the World Health Organization (WHO) updated its global guidance on PEP to include two new recommendations intended to increase timely access to and delivery of PEP. These recommendations specifically aim to expand both *where* PEP can be delivered, to include community settings, and *who* can provide PEP, to include community health workers and task‐sharing. The practical realities of adopting new public health guidelines to achieve the intended benefits in most contexts are complex. Articulating these realities is important for identifying what will be required to ensure the feasibility of expanded PEP access in community settings.

**Discussion:**

We provide stakeholder perspectives from five African countries—Kenya, Nigeria, South Africa, Uganda and Zimbabwe—on both barriers to and strategies for implementing the new WHO PEP recommendations. These perspectives are informed by experiences in these countries that were shared at a recent workshop and highlight key themes related to PEP uptake and use: awareness and acceptability; administration and monitoring; policy alignment, including regulatory considerations; logistics; integration of services; stakeholder involvement and capacity building; and linking PEP and PrEP more directly. Running across these themes are the roles of socio‐cultural norms and the need for increased resources to pay for implementing the recommendations, including capacity strengthening and monitoring in communities.

**Conclusions:**

While significant challenges exist to expanding PEP access in community settings and through task‐sharing, there are examples from our countries of successful efforts to mitigate them by leveraging existing community resources and capacities in innovative ways. Additional efforts will require engagement across multiple stakeholders to address remaining awareness gaps, logistical and regulatory obstacles, and political will. As countries work to update their guidelines and align with the new WHO recommendations, continued collaboration and innovation within and across countries will be essential to realize the full potential of PEP in comprehensive HIV prevention efforts.

## 1 INTRODUCTION

Despite encouraging reductions in HIV incidence across several countries, significant numbers of new transmissions persist, particularly among key and priority populations [1]. Even in settings where countries have achieved or are nearing the United Nations’ (UN) 95−95−95 targets, high levels of testing and antiretroviral therapy (ART) coverage have not been sufficient to achieve the low incidence rates necessary for epidemic control. This highlights the need for enhanced HIV prevention efforts and increased focus on optimizing the uptake of proven efficacious interventions.

The World Health Organization (WHO) recommends several interventions as part of a comprehensive HIV prevention strategy, including condom and lubricant programming, harm reduction for people who inject drugs, voluntary medical male circumcision, pre‐exposure prophylaxis (PrEP), provision of ART to eliminate vertical transmission of HIV and post‐exposure prophylaxis (PEP). Evidence indicates that providing a range of options and enabling individual choice is crucial for improving the uptake of HIV prevention interventions, which, in many contexts, remains low [2].

PEP, which involves taking antiretroviral medications shortly after potential exposure to HIV to reduce the risk of acquisition, is one such method with relatively low uptake. Although the evidence base for PEP as an efficacious HIV prevention method is limited, it has been accepted globally as sufficient to render PEP an important HIV prevention strategy for various types of exposure [3]. Even with that acceptance, access to and utilization of PEP remain suboptimal, even among healthcare workers in low‐ and middle‐income countries (LIMCs) where the risk of occupational exposure is high and among the general population in sub‐Saharan African countries with high HIV prevalence [4, 5, 6, 7].

In response to this gap—and building on the increased attention to and uptake of PrEP, which similarly involves taking antiretroviral medications, but in this case prior to HIV exposure—WHO updated its PEP guidelines in 2024. These guidelines aim to draw attention among policymakers, donors, programme managers, healthcare providers, communities and potential PEP users to the use of PEP. Specifically, these guidelines add two new recommendations: (1) expanding *where* PEP can be provided to include community settings, such as integrated community healthcare facilities and other, non‐healthcare facilities and services (e.g. pharmacies, community‐based organizations, drop‐in centres, mobile clinics and online delivery); and (2) expanding *who* can deliver PEP to include community‐based providers, such as pharmacists, nurses, doctors and trained lay and peer health workers, and to encourage task sharing among them [8].

These new recommendations are based on very limited evidence, which WHO acknowledges in both the main guidance document and the annex detailing its evidence review methodology [9]. Only 13 PEP‐specific studies related to the new recommendations met WHO's review criteria; about half of these were conducted in non‐African, non‐LIMCs (i.e. United States, United Kingdom, Canada, China). Other data used to support the recommendations came from studies of expanded delivery of antiretroviral drugs (ARVs) for HIV treatment and for PrEP. Notwithstanding this limited evidence, the Guidelines Development Group believed that the benefits of expanding PEP access in communities far outweighed any harms.

The practical realities of adopting new public health guidelines to achieve such benefits in most contexts are complex [10]. To ascertain the feasibility of implementing the two new PEP recommendations—especially given the limited evidence base supporting them—it is imperative to learn from country experiences with PEP delivery to date, and to identify existing and potential obstacles to and strategies for expanding PEP research, access and uptake in different settings.

## 2 DISCUSSION

In this commentary, we consider some existing barriers and potential solutions to PEP scale‐up within and across five countries: Kenya, Nigeria, South Africa, Uganda and Zimbabwe. Our discussion is informed by deliberations at a workshop held in June 2024, convened by the South‐to‐South HIV Prevention Learning Network (SSLN) with stakeholders from these five countries [11]. We focus on key, interrelated themes related to community provision of PEP and task‐sharing: awareness and acceptability; administration and monitoring; policy alignment; logistics; integration of services; stakeholder involvement and capacity building; and linking PEP and PrEP.

### 2.1 Awareness and acceptability

A significant challenge in PEP implementation is the widespread lack of awareness of PEP as an HIV prevention method among both healthcare providers and potential end‐users. Unpublished data from the Kenya Health Information System reported at the SSLN workshop revealed that between January 2022 and March 2024, less than 42% of young people in Kenya exposed to HIV presented for PEP within the required 72 hours due to fear and lack of knowledge. This awareness gap extends to healthcare providers, who often associate PEP primarily with sexual assault cases and occupational exposure. Thus, increasing the availability of PEP through community‐based delivery and task‐sharing requires increased awareness of PEP among both community members and healthcare staff.

In addition to limited awareness, stigma related to HIV and ARVs remains a substantial barrier to PEP uptake. Concerns about confidentiality and self‐stigma are significant challenges in implementing community‐based PEP delivery and task‐sharing.

To address these issues, we suggest several solutions that emerged from the SSLN workshop and resonate with our experiences, including provider training and sensitization on PEP guidelines; effective branding and repackaging of PEP to increase acceptability, especially among youth; using discreet packaging (e.g. zip lock envelopes or blister packs) to reduce stigma; incorporating recognizable and trusted figures for awareness campaigns; and ensuring the name and messaging (including the distinction between PEP and PrEP) are appealing and relevant to target populations. It is worth noting that, while *rebranding* PEP may be necessary for greater appeal, our consensus is that *renaming* PEP at the policy level is not. Rather, effective branding (e.g. how PEP drugs and their packaging look) and messaging to build awareness of PEP should be the focus of attention.

### 2.2 Administration and monitoring

Implementing the new recommendations requires careful consideration of key aspects of PEP administration and monitoring, including HIV testing, PEP prescribing and dosing, and adherence counselling and support inclusive of managing commonly perceived and experienced negative side effects of PEP medications [7]. A broader array of PEP providers will have to be trained in these areas; how such expanded training and retraining will be conducted and funded is a concern. Notwithstanding, countries are moving forward with innovations in these areas. In Uganda, for example, community health workers (CHWs) are being trained to support adherence monitoring and conduct HIV self‐testing both pre‐ and post‐PEP, which enables expanded service coverage without overburdening healthcare infrastructure. Additionally, “PEP on Demand” and mobile delivery models are being piloted in South Africa to ensure timely access to PEP even in remote or resource‐limited areas. Peer‐led adherence support and the introduction of mobile clinics also demonstrate promising results in increasing adherence and providing follow‐up support, particularly among youth [11].

### 2.3 Policy alignment

Expanding PEP access requires policy changes and considerations. These include updating local and national policies to align with the new WHO recommendations; conducting comprehensive budget analyses for expanding PEP in community settings; addressing regulatory restrictions on where PEP and associated HIV testing can be delivered and by whom, as well as on which drugs can be used for PEP; and updating healthcare workers’ scopes of work.

Relevant extant policies are reflected not only in PEP‐specific guidelines and strategies, but also in those addressing the use of ART for both prevention (including PrEP) and treatment [12]. Ideally, such guidelines should be merged and harmonized and should clearly define PEP's role within broader HIV prevention strategies. This requires leveraging existing structures and political will, which may be challenging.

Even with such policy and regulatory challenges, some countries are moving forward. For example, in Nigeria, regulatory requirements have limited PEP delivery to clinical settings, creating access bottlenecks. Policymakers there are working to amend guidelines to authorize community provision and promote PEP as a tool for all high‐risk exposures, not only occupational or sexual assault‐related cases. Kenya and Uganda also are revising their policies to broaden PEP's availability through CHWs, private pharmacies and drop‐in centres, expanding PEP's reach in urban and rural communities alike.

### 2.4 Logistics

Effective implementation of community‐based PEP requires addressing logistical challenges, including forecasting demand and commodity needs and managing supply chain factors such as procurement, logistics, stock monitoring and distribution, which differ in rural and urban/peri‐urban settings. Uganda and Kenya have experienced issues related to stock monitoring and supply chain inefficiencies, which can result in stock‐outs at community distribution points.

We suggest establishing diversified PEP delivery points—such as youth centres, private pharmacies and mobile clinics—and leveraging digital tools to track inventory and monitor demand. As an example, South African mobile clinics and CHWs now carry small PEP stockpiles, allowing immediate dispensation upon need without requiring patients to travel to centralized health facilities. Further piloting PEP delivery through various channels, including pharmacies, online platforms and vending machines, can improve accessibility.

### 2.5 Integration of services

The integration of PEP with other HIV, sexually transmitted infections (STIs), and sexual and reproductive health (SRH) services is crucial for expanding access. This requires leveraging existing community structures for the delivery of multiple prevention methods, including HIV testing/self‐testing, and strengthening referral systems between health services.

Nigeria has integrated and trained CHWs for task shifting and sharing along the continuum of care in its HIV treatment and care guidelines. Kenya and Uganda are pursuing integration strategies to link PEP services with STI treatment, emergency contraception and HIV self‐testing kits, making PEP more accessible and comprehensive within existing health services. Zimbabwe has adopted a community health approach that integrates PEP into SRH and family planning services within safe spaces, youth drop‐in centres and primary healthcare clinics. This one‐stop‐shop model allows for seamless transitions from emergency PEP use to other preventive measures like PrEP, supporting continuous care for at‐risk populations.

### 2.6 Stakeholder involvement and capacity building

Successful implementation of the new WHO recommendations requires engaging a wide range of stakeholders and building capacity among healthcare workers and community providers to raise awareness, drive PEP adoption and address cultural barriers. Key aspects of engagement include involving private sector actors, policy implementers, law enforcement agencies, community, traditional and religious leaders; building healthcare workers’ capacity to deliver PEP effectively and sensitively; incorporating PEP into HIV prevention demand creation strategies; and addressing social norms and stigma to build community support.

There are examples of efforts in this direction from our countries. In Nigeria, local radio stations and community influencers have been engaged to raise awareness and promote acceptance of PEP; and in Zimbabwe, healthcare providers are receiving training to increase empathy and reduce judgement and stigma towards individuals eligible for PEP. Political support has proven a vital facilitator, as seen in Uganda, where government leaders endorsed PEP as part of the national HIV prevention agenda, opening the door to policy changes that support community‐based PEP access. The private sector also plays a pivotal role, with pharmacies in Kenya and South Africa serving as alternative PEP access points, thus decentralizing its provision and increasing accessibility.

In addition to these efforts, we need enhanced monitoring, evaluation and learning, including developing partnerships for community‐led monitoring (CLM) [13], among key populations, to ensure services are acceptable and effective.

### 2.7 PEP and PrEP linkages

The aim of integrating PEP and PrEP within HIV prevention frameworks and strategies presents both significant opportunities and challenges. By linking these prevention tools, healthcare systems can provide individuals with a more comprehensive HIV prevention pathway, allowing for seamless transitions from emergency PEP usage to ongoing PrEP when appropriate. However, operationalizing this integration in the context of the new WHO recommendations and the emergent access to long‐acting injectable PrEP requires adjustments in all the thematic areas discussed above.

One primary challenge is aligning national prevention guidelines with updated WHO recommendations that define PEP's role within broader HIV prevention strategies. Many existing national policies restrict PEP provision to occupational or sexual assault cases, limiting its use as a preventive measure. Another challenge is the lack of clarity in current guidelines about the transition from PEP to PrEP, particularly in settings where different healthcare providers handle each service. For effective implementation, guidelines must streamline the clinical decision‐making process for healthcare workers, detailing when and how to introduce PrEP to individuals completing a PEP course.

To bridge these policy gaps, we suggest integrating WHO guidelines into country‐specific policies to clearly define the role of both PEP and PrEP. Our recommendations include updating national protocols to account for the unique needs of different risk profiles and emphasizing patient/user choice. This would involve updating standard operating procedures and prescription protocols to guide healthcare workers in supporting the effective transition from PEP to PrEP. MOSAIC has developed an adaptable PEP template guidelines for countries to review, develop and adapt their existing PEP guidelines to ensure alignment with the new recommendations [14]. The template guideline can be included into the existing PEP guidelines or as an addendum, depending on the country needs.

Training CHWs and other non‐traditional providers, such as pharmacists, to dispense PEP and counsel on PrEP offers a pathway to increase coverage and reduce the burden on higher‐level healthcare facilities. Uganda and Kenya are already pursuing these strategies, working to integrate CHWs into the continuum of care for PEP and PrEP services. Training modules that cover transition protocols, risk assessment and culturally sensitive communication techniques are crucial. Creating user‐friendly risk assessment tools, both digital and manual, can also support healthcare workers in guiding patients through the transition from PEP to PrEP.

Finally, it is important to highlight the role of monitoring and evaluation in ensuring effective PEP‐PrEP linkages through enhancing monitoring, evaluation and learning systems, including developing indicators and tools that capture client/user feedback on both interventions; strengthening pharmacovigilance and drug resistance monitoring systems for both PEP and PrEP; and employing CLM for accountability. These measures would help track the success of linkage efforts and identify areas for improvement.

## 3 CONCLUSIONS

While there is a pressing need to revitalize PEP as a crucial component of HIV prevention, significant challenges remain in practical implementation, particularly in rolling out PEP in community settings and task‐sharing. Expanding timely PEP access will require engagement across multiple stakeholders to meaningfully address awareness gaps, logistical and regulatory obstacles, and political will. It will require leveraging existing community resources and strengths to optimize local solutions. And it will require support for context‐specific research and advocacy to ensure that expanded delivery channels provide options and choices to people. Our commentary highlights some good examples of efforts in these directions, but more clearly must be done. As national public health programmes work to update their guidelines and align with the new WHO recommendations, continued collaboration and innovation within and across countries will be essential to realize the full potential of PEP in comprehensive HIV prevention efforts.

## COMPETING INTERESTS

All authors declare that they have no competing interests.

## AUTHORS’ CONTRIBUTIONS

SM conceptualized the commentary and provided initial notes. JDA developed the first draft of the manuscript with significant inputs from SM. DB, MSH, JM and IM contributed additional inputs to the draft manuscript. All authors reviewed, edited and approved the final manuscript.

## FUNDING

JDA's work on the manuscript was supported by the Bill & Melinda Gates Foundation, contract #INV‐053199. SM's work on the manuscript was supported by the Bill & Melinda Gates Foundation, contract #INV‐073717 .

## Data Availability

Data sharing is not applicable to this article as no new data were created or analyzed in this study.
